# Prediction, Detection, and Validation of Isotope Clusters in Mass Spectrometry Data

**DOI:** 10.3390/metabo6040037

**Published:** 2016-10-20

**Authors:** Hendrik Treutler, Steffen Neumann

**Affiliations:** 1 Department of Stress and Developmental Biology, Leibniz Institute for Plant Biochemistry, Weinberg 3, Halle 06120, Germany; steffen.neumann@ipb-halle.de; 2 Institute of Computer Science, Martin-Luther-University Halle-Wittenberg, Von-Seckendorff-Platz 1, Halle 06120, Germany

**Keywords:** isotope cluster, software, raw data

## Abstract

Mass spectrometry is a key analytical platform for metabolomics. The precise quantification and identification of small molecules is a prerequisite for elucidating the metabolism and the detection, validation, and evaluation of isotope clusters in LC-MS data is important for this task. Here, we present an approach for the improved detection of isotope clusters using chemical prior knowledge and the validation of detected isotope clusters depending on the substance mass using database statistics. We find remarkable improvements regarding the number of detected isotope clusters and are able to predict the correct molecular formula in the top three ranks in 92% of the cases. We make our methodology freely available as part of the Bioconductor packages *xcms* version 1.50.0 and *CAMERA* version 1.30.0.

## 1. Introduction

The elucidation of the metabolism provides deep insights into complex processes in the cell such as responses to nutrition deficiency, pathogen exposure, and drought stress in plants or the implications of mutations, age, and tissue development in animals. Mass spectrometry is a key technology for the identification and quantification of metabolites in biological samples. After measurement using mass spectrometers, feature detection algorithms extract basic properties about peaks in the raw data such as retention time and peak height. The set of properties describing single peaks are called *features* and the exhaustive extraction of features is a prerequisite for downstream analyses such as metabolite identification and quantitative comparisons between samples.

The feature detection algorithm *centWave* in the R package *xcms* version 1.50.0 [1] adapts the following procedure. First, a set of *regions of interest* (ROIs) is identified in the ROI identification step, where ROIs are two-dimensional intervals in the mass-to-charge (*m*/*z*) dimension and the retention time dimension containing potential signals. The set of ROIs is examined in the ROI examination step in order to validate, localize, and quantify features. In the ROI identification step, a heuristic method is applied to the raw data to substantially reduce the processing time of the more computationally intensive ROI examination step. This heuristic method aims at a high specificity at the cost of sensitivity, especially in case of features with a low signal-to-noise ratio. Consequently, potentially important features in the raw data are not detected and the information behind these features cannot be used in downstream analyses.

Most chemical elements are present in different variants called isotopes. Though chemically almost equivalent, the isotopes of a particular chemical element differ in mass and are thus well distinguishable using mass spectrometry. The isotopes of each element have a known natural abundance and the distribution of isotopes across all atoms of a molecule results in a set of related signals. The features extracted from these signals are called *isotopologue features* and the set of all isotopologue features from one analyte is called *isotope cluster* also known as isotope pattern. Unfortunately, many of these signals are below the detection limit which results in the underestimation of isotopologue features.

Based on isotope clusters, it is possible to determine the charge state, abundance, and elemental composition of the measured ion with high precision. The arrangement of isotopologue features to isotope clusters leads to a considerable reduction of data complexity facilitating the interpretation of data sets. It has been demonstrated that the analysis of isotope clusters leads to an increased confidence and precision of comparative analyses [2]. Isotope clusters from precursor ions and tandem mass spectrometry are pivotal for the determination of the molecular formula using software like SIRIUS [3], Rdisop [4], and others [5,6,7,8,9,10,11,12]. The molecular formula strongly facilitates the identification of molecules known as a major bottleneck in metabolomics [13,14] and has been demonstrated metabolome-scale [15]. There are approaches in metabolomics and proteomics which use isotope clusters to improve peak picking [16,17,18]. In addition, isotope clusters have been used as a valuable source for the assessment of the data quality [19] and for database searches with high precision [20].

The detection of isotope clusters is usually performed after peak picking by consideration of coeluting features separated by certain distances in the *m*/*z* dimension. However, a validation of putative isotope clusters in terms of the removal of leading peaks from hydrogen–losses and the decomposition of overlapping isotope clusters into individual isotope clusters is usually lacking in case of small molecules. The deconvolution of overlapping isotope clusters has been described in case of peptides and proteins, for isotope dilution experiments, and in case of substances with known molecular formula [17,21,22].

Aiming at the exhaustive detection and precise validation of isotope clusters, we propose the following approach for liquid chromatography–high resolution mass spectrometry data. We predict new ROIs for putative isotope peaks based on previously detected features and implement this approach in combination with the *centWave* algorithm as part of the R package *xcms* version 1.50.0 [23]. We validate putative isotope clusters depending on the mass of the substance based on database statistics and implement this approach as part of the R package *CAMERA* version 1.30.0 [24].

For evaluation purposes, we apply the modified *centWave* algorithm to different sets of mass spectrometry raw data and detect and validate isotope clusters as proposed. We evaluate the results using various performance measures and find remarkable improvements regarding the number of detected isotope clusters. The extended R packages *xcms* and *CAMERA* are available at Bioconductor [25].

## 2. Results

We demonstrate the performance of our approach for an enhanced isotope cluster detection and validation. First, we describe the workflow which includes our approach; Second, we evaluate the proposed targeted peak picking with predicted isotope ROIs compared to peak picking with random ROIs and traditional peak picking on basis of various performance measures; Third, we evaluate the proposed isotope detection routine with mass–specific isotope cluster validation compared to several isotope detection routines on basis of various performance measures; Fourth, we present the isotope ratio quantiles which are used for the validation of isotope clusters; Fifth, we exemplify the proposed isotope detection routine with and without mass–specific isotope cluster validation on six example substances.

### 2.1. Workflow of the Approach

**Figure 1:**
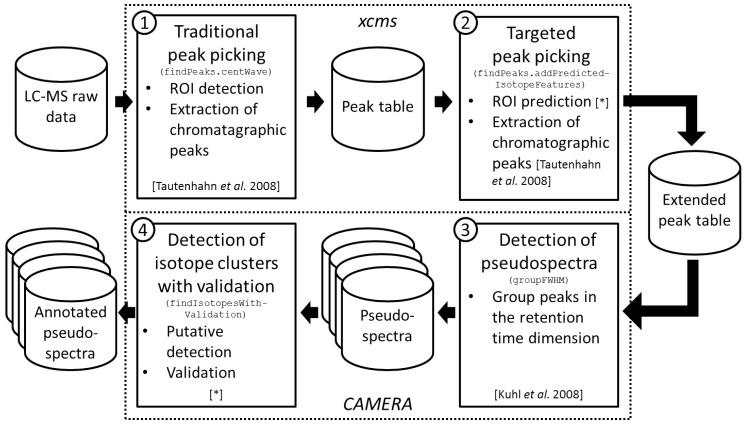
Workflow of the proposed approach. We depict data sets with cylinders, algorithms with continuous rectangles, and R packages with dotted rectangles. Each algorithm rectangle comprises the step number (top left corner), the purpose of the algorithm (heading), the R function name (monospace font), algorithm steps (itemized), and a reference for the algorithm or the individual algorithm steps (in square brackets, asterisk stands for this manuscript). ① The workflow starts with traditional peak picking on LC-MS raw data to extract a peak table comprising features; ② This peak table is extended by a targeted peak picking which targets on isotope features; ③ The extended peak table is split into putative compound spectra denoted pseudospectra; ④ The detection and validation of isotope clusters is performed on each pseudospectrum resulting in annotated pseudospectra.

We integrated the proposed methodology into an untargeted workflow which extracts annotated peak tables from LC-MS raw data as summarized in Figure 1. The user supplies the LC-MS raw data files in a *xcms*-supported format, namely one of AIA/ANDI NetCDF, mzXML, mzData, or mzML. The workflow incorporates one function from the R package *xcms* [23], one function from the R package *CAMERA* [24], and two new function as follows.

First, we perform peak picking without any prior knowledge which we denote as *traditional peak picking*. Here, we use the *centWave* algorithm [1] which applies a heuristic for the detection of ROIs (ROI identification step). Given the set of detected ROIs, chromatographic peaks are extracted using continuous wavelet transformation (ROI examination step). This step results in a peak table with one row for each detected feature and one column for each feature property such as *m*/*z*, retention time, integrated peak area, and signal-to-noise ratio.

Second, we perform the proposed targeted peak picking as described in Section 4.1. Here, a set of isotope ROIs is predicted on basis of the previously extracted peak table. Given the set of predicted isotope ROIs, chromatographic peaks are extracted using continuous wavelet transformation (ROI examination step). Notably, this ROI examination step is identical to the ROI examination step in the traditional peak picking step with the exception that we use relaxed peak picking parameters this time. This step results in an extended peak table which is enriched with features corresponding to isotope isotope peaks as demonstrated in the second results section.

Third, we extract *pseudospectra* from the extended peak table [24]. This step aims at the extraction of compound spectra on basis of the retention times, but multiple coeluting compounds are potentially assigned to the same spectrum which is the reason for the usage of the term pseudospectrum. In case of multiple raw data files a retention time correction (*xmcs* function retcor) can be advisable prior to the extraction of pseudospectra. This step results in a set of pseudospectra. Each pseudospectrum is a peak table comprising all properties of a subset of the features from the extended peak table.

Fourth, we detect isotope clusters in each pseudospectrum using the proposed isotope detection routine with mass–specific isotope cluster validation as described in Section 4.2. Here, putative isotope clusters are detected and putative isotope clusters are validated based on database statistics as demonstrated in the third results section. This step results in a set of annotated pseudospectra, i.e., the given set of pseudospectra enriched with isotope annotations.

The presented workflow is implemented exemplarily in the vignette IsotopeDetectionVignette in R package *CAMERA* in version 1.30.0. In addition the R package *CAMERA* supports a number of further analyses given the set of annotated pseudospectra. This includes, amongst others, the annotation of adducts and neutral losses, the filling of missing values, and the combination of results from opposite ion modes.

### 2.2. Targeted Peak Picking Using Predicted Isotope ROIs

We examine whether the proposed prediction of isotope ROIs in combination with the *centWave* algorithm increases the number of detected isotope peaks. To verify the specificity of the predicted isotope ROIs to isotopes, we compare predicted isotope ROIs with the same number of random ROIs denoted *noise ROIs*. In addition, we compare our approach to the unmodified *centWave* algorithm with different signal-to-noise thresholds snthr. We evaluate our approach based on a dilution series experiment with 40 LC-MS measurements. These data sets comprise both strong and weak signals and constitute the basis to test the detection of weak signals like isotope peaks.

We evaluate the performance of predicted isotope ROIs detected with different relaxed signal-to-noise thresholds snthr’ as described in Section 4.1 on 40 LC-MS measurements described in Section 4.4. We quantify the performance using the performance measures (i) number of detected peaks; (ii) number of detected isotope peaks; (iii) number of detected isotope clusters; (iv) *isotope coverage*; and (v) Peak Picking Score (*PPS*). The isotope coverage is the ratio between the number of detected isotope peaks and the number of detected peaks. The isotope coverage ranges from 0 to 1, where 0 means that no isotope clusters have been detected and 1 means that all peaks are part of isotope clusters. A higher isotope coverage indicates a higher peak picking quality as exploited in [19]. The PPS was proposed in [19] for the quantification of the peak picking quality and implemented in the R package *IPO*. The PPS is defined as the ratio between the number of reliable peaks squared and the number of non–reliable peaks. The number of reliable peaks is defined as the number of peaks in isotope clusters which are detected in the *IPO* package by a custom isotope detection routine. The number of non–reliable peaks is defined as the number of peaks which are not in a isotope cluster although it is to be expected based on different criteria. We compute each performance measure as a function of the relaxed signal-to-noise threshold ${\mathrm{snthr}}^{\prime }\;\in \left\{100,95,...,5\right\}\%\;*\;\mathrm{snthr}$, where snthr=25 is the signal-to-noise threshold used in the traditional peak picking step.

**Figure 2:**
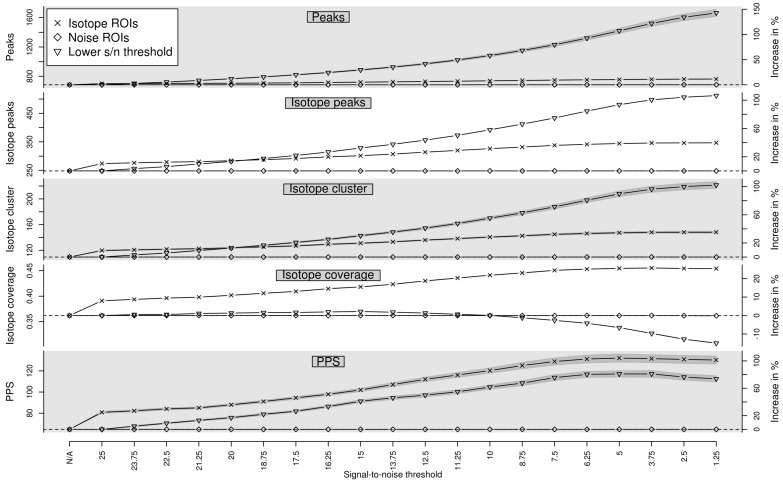
Evaluation of predicted isotope ROIs for varying relaxed signal-to-noise threshold snthr’. We show the mean (solid line) and the standard error of the mean (SEM, interval in dark grey) of the performance measures (**i**) number of detected peaks; (**ii**) number of detected isotope peaks; (**iii**) number of detected isotope clusters; (**iv**) isotope coverage; and (**v**) Peak Picking Score (PPS). In case of isotope ROIs and noise ROIs, we plot the performance of each measure without additional ROIs in the first column (“N/A”) as reference value (horizontal dashed line) and in the subsequent columns with additional ROIs for decreasing relaxed signal-to-noise threshold snthr’. In case of “Lower S/N threshold”, we plot the performance of each measure for decreasing signal-to-noise threshold snthr without additional ROIs. All four measures increase for predicted isotope ROIs with decreasing signal-to-noise threshold snthr’ in contrast to noise ROIs.

In Figure 2 we show the performance of the traditional peak picking in combination with targeted peak picking with isotope ROIs as well as traditional peak picking in combination with targeted peak picking with noise ROIs for varying signal-to-noise threshold snthr’. In addition, we show the performance of traditional peak picking with varying signal-to-noise threshold snthr. In case of predicted isotope ROIs, all five measures increase with decreasing snthr’. The isotope coverage appears to saturate for a relaxed signal-to-noise threshold snthr’ of approximately 6.25. For this threshold, we find in case of predicted isotope ROIs an average increase of approximately +10% peaks, +37.6% isotope peaks, +33.5% isotope clusters, +25.2% isotope coverage, and +102.8% PPS in contrast to noise ROIs, suggesting an isotope-specific improvement of peak picking. More specifically, 20 isotope clusters could be extended and 37 isotope clusters could be newly detected. In addition, we find that the PPS decreases for a relaxed signal-to-noise threshold snthr’ lower than 5. This finding confirms the general observation that peak picking with a too low signal-to-noise threshold results in unreliable peaks and is therefore not advisable. We also tested the performance of traditional peak picking with varying signal-to-noise threshold snthr and find that the number of peaks more than doubles. However, the proportion of low–intensity peaks which are not part of isotope clusters increases disproportionately and there is no specificity for isotope peaks.

### 2.3. Isotope Cluster Detection and Validation

**Figure 3:**
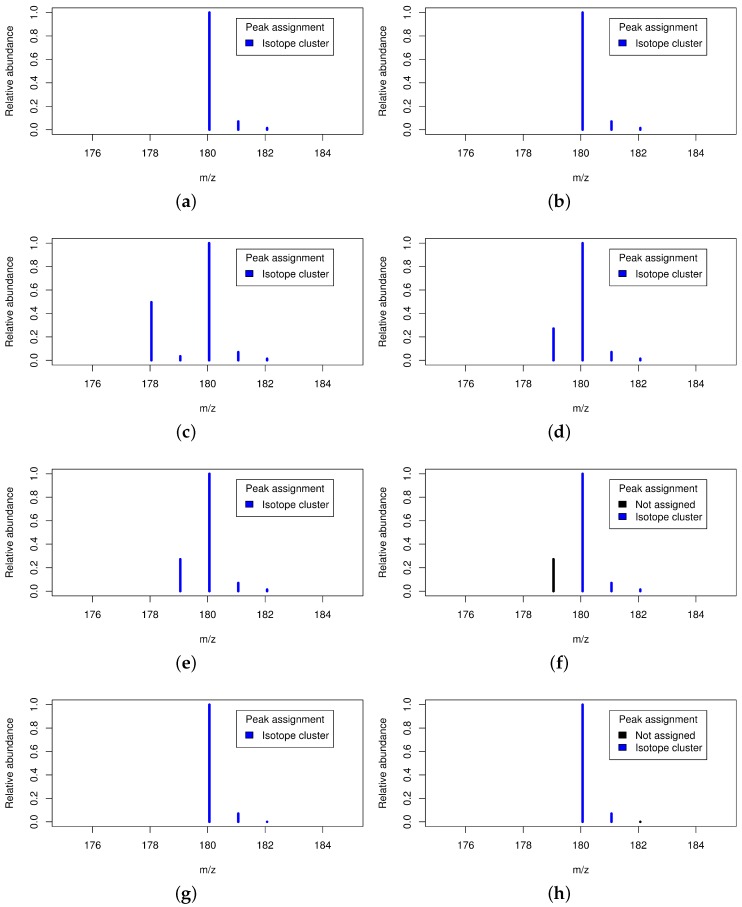
Four cases necessitating the validation of putative isotope clusters. Figure 3**a,b**: Valid isotope cluster without and with isotope cluster validation; Figure 3**c,d**: Two overlapping isotope clusters without and with isotope cluster validation; Figure 3**e,f**: Hydrogen loss without and with isotope cluster validation; Figure 3**g,h**: Underestimated small peak without and with isotope cluster validation.

There is a multitude of isotope detection routines for the recognition of isotope clusters. These detect coeluting features which are separated by certain distances in the *m*/*z* dimension and group these features to isotope clusters. However, a validation of detected isotope clusters is typically based on simple *ad hoc* rules. There are at least four cases for which the validation of isotope clusters can be beneficial as shown in Figure 3.

First, valid isotope clusters can be verified which strengthens the trust in the data; Second, multiple coeluting substances with mass differences of a few dalton can result in isobaric ion species and thus in overlapping isotope clusters [26]. These are potentially misinterpreted as a single isotope cluster affecting downstream analyses. This necessitates the deconvolution of the overlapping isotope cluster into at least two valid isotope clusters; Third, substances can be affected by hydrogen loss as reported in [27] and exploited in [28]. This leads to mass differences similar to isotope peaks ($\mathrm{mass}{(}^{1}\text{H})$ = 1.008 ≈ 1.0034 = mass${(}^{13}\text{C})$ − mass${(}^{12}\text{C})$) and results in a small trailing peak which is potentially misinterpreted as monoisotopic peak of the putative isotope cluster. This may result in the assumption of a wrong monoisotopic mass and may even lead to the rejection of the entire isotope cluster on the basis of failed intensity-checks [24]. Although this small trailing peak corresponds to the same substance, it needs to be removed from the isotope cluster in order to allow more precise molecular formula predictions. Fourth, the intensity of small peaks is systematically underestimated by some mass spectrometers which leads to distorted ratios between different isotope peaks as reported previously [3]. This intensity bias would lead to distorted molecular formula predictions and the removal of these underestimated peaks from the isotope cluster allows more precise molecular formula predictions.

We compare the proposed isotope detection routine with mass–specific isotope cluster validation (${\mathrm{IDR}}_{\mathrm{NewVal}}$) with the isotope detection routine without isotope cluster validation (${\mathrm{IDR}}_{\mathrm{NewNoVal}}$), the isotope detection routine implemented in the *AStream* package (${\mathrm{IDR}}_{\mathrm{AStream}}$) [29], the isotope detection routine implemented in the *CAMERA* package (${\mathrm{IDR}}_{\mathrm{CAMERA}}$) [24], and the isotope detection routine implemented in the *mzMatch* package (${\mathrm{IDR}}_{\mathrm{mzMatch}}$) [30]. The isotope detection routines from *AStream*, *CAMERA*, and *mzMatch* apply different requirements for the validation of isotope clusters. In ${\mathrm{IDR}}_{\mathrm{AStream}}$ it is required that the abundance of the monoisotopic peak, the first isotope peak, and the second isotope peak decreases strictly, which corresponds to a ratio <1 between consecutive isotope peaks. In ${\mathrm{IDR}}_{\mathrm{CAMERA}}$ it is required that the ratio of the monoisotopic peak to the first isotopic peak is within an interval which is given by the ratios of the monoisotopic peak to the first isotopic peak of a substance consisting exactly one carbon atom and a substance consisting exactly ${\mathrm{mass}}_{\mathrm{mono}}{/\mathrm{mass}(}^{12}\text{C})$ carbon atoms, where ${\mathrm{mass}}_{\mathrm{mono}}$ is the assumed monoisotopic mass of the substance. In ${\mathrm{IDR}}_{\mathrm{mzMatch}}$ it is required that isotope peaks show a high correlation regarding coelution.

**Figure 4:**
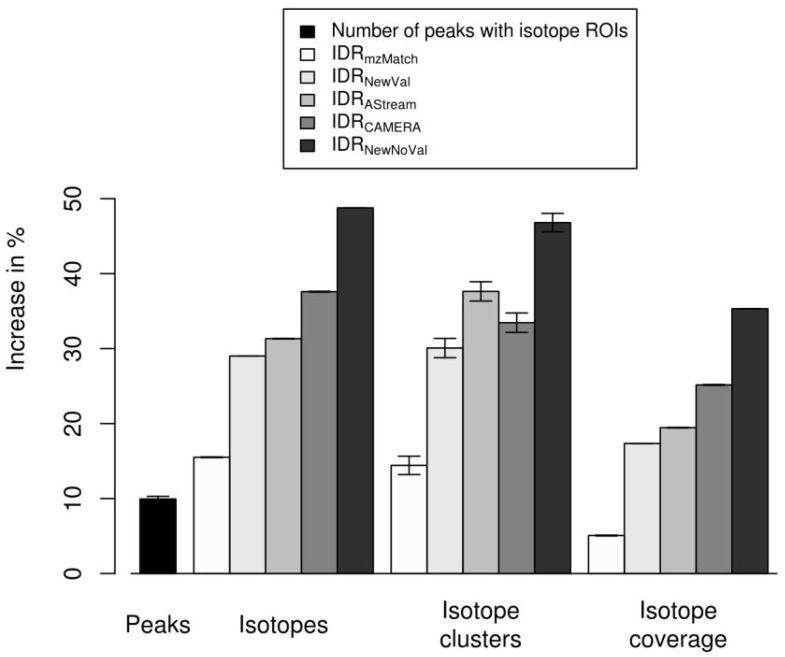
Evaluation of predicted isotope ROIs in combination with different isotope detection routines for a relaxed signal-to-noise threshold snthr’ of 6.25. We plot the increase of the mean and the standard error of the mean (SEM, error bars) of the performance measures (**i**) number of detected peaks; (**ii**) number of detected isotope peaks; (**iii**) number of detected isotope clusters; and (**iv**) isotope coverage relative to the performance of the *CAMERA* isotope detection routine without predicted isotope ROIs. All four measures increase with predicted isotope ROIs.

**Figure A1:**
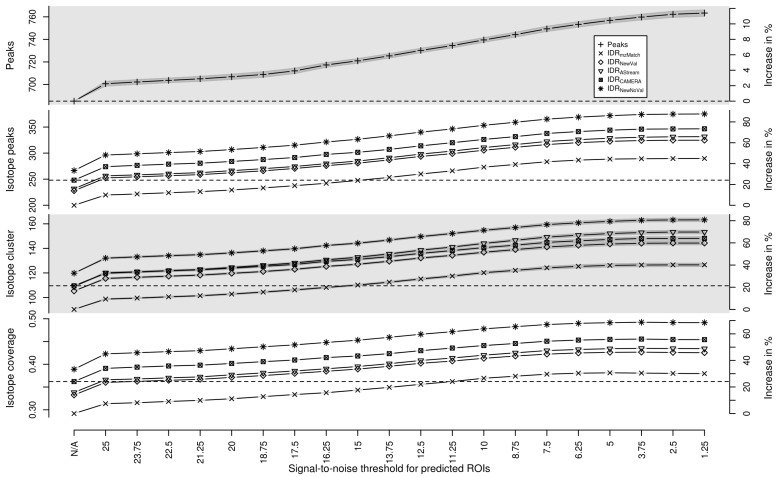
Evaluation of predicted isotope ROIs in combination with validated isotope clusters for varying relaxed signal-to-noise threshold snthr’. We plot the mean (solid line) and the standard error of the mean (SEM, interval in dark grey) of the performance measures (**i**) number of detected peaks; (**ii**) number of detected isotope peaks; (**iii**) number of detected isotope clusters; and (**iv**) isotope coverage. We plot the performance of each measure without additional ROIs in the first column (“N/A”) as reference value (horizontal dashed line). All four measures of all isotope detection routines increase with decreasing signal-to-noise threshold snthr’.

We evaluate the performance of the isotope cluster detection and validation described in Section 4.2 on a dilution series experiment with 40 LC-MS measurements described in Section 4.4. We quantify the performance using the performance measures (i) number of detected peaks; (ii) number of detected isotope peaks; (iii) number of detected isotope clusters; and (iv) isotope coverage, i.e., the proportion of detected isotope peaks versus all detected peaks. We compute each performance measure without predicted isotope ROIs as well as with predicted isotope ROIs for a relaxed signal-to-noise threshold snthr’ of 6.25. We present the results with predicted isotope ROIs relative to the results without predicted isotope ROIs in Figure 4. These results are a subset of the results in Figure A1 in the Appendix A where we present the results for varying relaxed signal-to-noise threshold snthr’. We relate the results to the quality of the predicted molecular formulas presented in the Appendix B on a gold standard of 11 data sets with known content.

In Figure 4 we show the performance measures for ${\mathrm{IDR}}_{\mathrm{NewVal}}$, ${\mathrm{IDR}}_{\mathrm{NewNoVal}}$, ${\mathrm{IDR}}_{\mathrm{AStream}}$, ${\mathrm{IDR}}_{\mathrm{CAMERA}}$, and ${\mathrm{IDR}}_{\mathrm{mzMatch}}$. We find that all four measures increase with predicted isotope ROIs in case of all isotope detection routines. ${\mathrm{IDR}}_{\mathrm{NewNoVal}}$ detects the most isotopes which reflects the fact that there are no constraints regarding the shape of the isotope cluster. This indicates that a certain proportion of the detected isotope clusters might be invalid. We point out, that this highly sensitive algorithm can be useful in case of substances containing uncommon elements such as Cl, Br, Se, or B as scrutinized in [31]. ${\mathrm{IDR}}_{\mathrm{mzMatch}}$ detects by far the lowest number of isotopes which reflects that this algorithm requires a high degree of correlation between isotope peaks resulting in a high specificity at the cost of sensitivity. ${\mathrm{IDR}}_{\mathrm{NewNoVal}}$ and ${\mathrm{IDR}}_{\mathrm{mzMatch}}$ show the lowest number of correctly predicted molecular formulas as shown in Appendix B. We find comparable results for ${\mathrm{IDR}}_{\mathrm{AStream}}$, ${\mathrm{IDR}}_{\mathrm{CAMERA}}$, and ${\mathrm{IDR}}_{\mathrm{NewVal}}$. Also the numbers of correctly predicted molecular formulas are similar as shown in Appendix B. Interestingly, ${\mathrm{IDR}}_{\mathrm{NewVal}}$ showed the highest number of correctly predicted molecular formulas and was also able to rank the highest number of correct molecular formulas to the first three ranks. Remarkably, in case of 85% to 92% of all tested ions the detected isotope clusters from all isotope detection routines with or without predicted isotope ROIs were sufficient for the prediction of the correct molecular formula to the first three ranks. This finding states, that the prediction of molecular formulas from isotope clusters works well in general and hence it is challenging to improve upon.

### 2.4. Isotope Cluster Statistics

We examine the compounds of the publicly available databases ChEBI [32], KEGG [33], KNApSAcK [34], LIPID MAPS [35], and PubChem [36] in order to compute mass–specific confidence intervals for the abundance–ratio of the monoisotopic peak to the first to fifth isotope peak as described in Section 4.3. For each database and each isotope peak, we compute multiple quantiles in order to define confidence intervals with different confidence levels. We validate isotope clusters on basis of mass–specific confidence intervals of peak abundance–ratios as described in Section 4.2.

We exemplarily examine the interval size and magnitude of the computed confidence intervals of isotope ratios. A small interval size indicates a small range of observed isotope ratios for the analyzed substances and allows a precise definition of valid isotope ratios, whereas a large interval size indicates a diverse range of observed isotope ratios for the analyzed substances and requires a loose definition of valid isotope ratios. If the interval size and magnitude of the computed confidence intervals depends on the mass range, then mass–specific confidence intervals can increase the specificity of isotope cluster validation.

**Figure 5:**
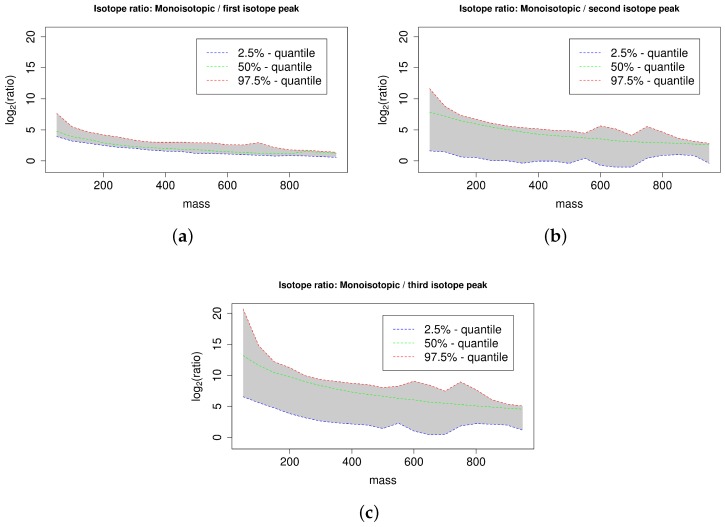
95% confidence interval of the ratio of the monoisotopic peak to the first (**a**), second (**b**), and third isotopic peak (**c**) of all compounds in KEGG for different compound masses arranged in mass windows of size 50 dalton. We plot the 50%-quantile in green, the 2.5%-quantile in blue, and the 97.5%-quantile in red and we emphasize the enclosed 95% confidence interval in grey. The ratios decrease with increasing compound mass reflecting the increasing proportion of isotopic atoms.

**Figure C1:**
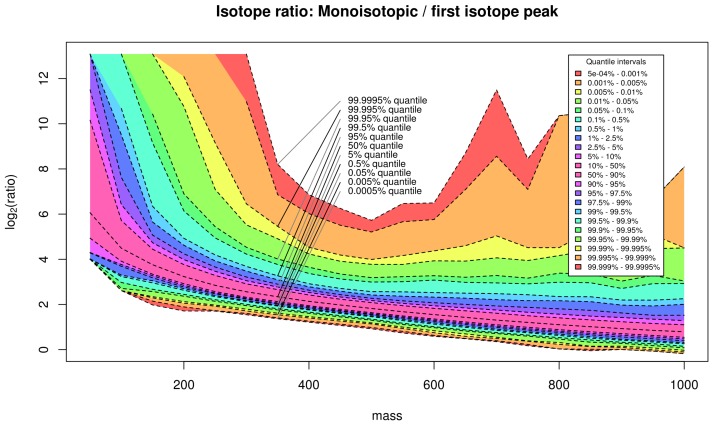
The full set of 23 quantiles of the monoisotopic peak versus the first isotopic peak for the PubChem database for different compound masses arranged in mass windows of size 50 dalton. We emphasize the enclosed confidence intervals with different colors.

See Figure 5 for the 95% confidence interval of the ratios of the monoisotopic peak to the first; second, and third isotope peak for the database KEGG with a mass window size of 50 dalton. The ratio of the monoisotopic peak to the first isotope peak depends on the abundance of the first isotope peak, which is dominated by the proportion of ^13^C. This results in a relatively narrow confidence interval, because the variation of the number of carbon atoms is limited within a 50 dalton mass window. The ratio of the monoisotopic peak to the second isotope peak depends on the abundance of the second isotope peak, which is dominated by the proportion of ^13^C and ^34^S. The 97.5%-quantile and the 50%-quantile are higher compared to the case of the first isotope peak because the second isotope peak has typically a lower abundance than the first isotope peak. In contrast, the 2.5%-quantile is smaller compared to the case of the first isotope peak because a subset of compounds comprises at least one sulfur (partially also chlorine or bromine) with a high abundance of ^34^S (or ^37^Cl, ^81^Br) causing a relatively high abundance of the second isotope peak and thus a small ratio of the monoisotopic peak to the second isotope peak. This results in a relatively large confidence interval. The ratio of the monoisotopic peak to the third isotope peak mainly depends on the abundance of the third isotope peak, which is dominated by the proportion of ^13^C and ^34^S (and ^37^Cl, ^81^Br). This results in a relatively large confidence interval analogous to the case of the second isotope peak. The quantiles are higher compared to the case of the second isotope peak because the third isotope peak has typically a lower abundance compared to the second isotope peak. We find that the magnitude of the quantiles substantially depends on the mass of the substances. Specifically, the quantiles are typically inversely proportional to the substance mass. For example, in case of the mass interval 200 to 250 dalton versus the mass interval 800 to 850 dalton the 50%-quantiles deviate by a factor of 3.5 in case of the ratio of the monoisotopic peak to the first isotope peak, by a factor of 8.4 in case of the ratio of the monoisotopic peak to the second isotope peak, and by a factor of 25.6 in case of the ratio of the monoisotopic peak to the third isotope peak. This finding suggests that mass–specific confidence intervals can indeed increase the specificity of isotope cluster validation. See Figure C1 in Appendix C for an overview of all computed quantiles and the resulting symmetric confidence intervals of the ratio of the monoisotopic peak to the first isotope peak for the database PubChem with a mass window size of 50 dalton.

### 2.5. Exemplary Isotope Cluster Detection

We exemplify the detection of isotope clusters for selected substances to demonstrate the proposed isotope detection routine without isotope cluster validation ${\mathrm{IDR}}_{\mathrm{NewNoVal}}$ and the isotope detection routine with mass–specific isotope cluster validation ${\mathrm{IDR}}_{\mathrm{NewVal}}$. We simulate the mass and relative intensity of the monoisotopic peak and the first five isotope peaks of six substances with enviPat [37] in centroid mode with a resolution of 10,000, namely (i) aspartic acid which has a low mass and comprises only the elements CHNO (see Table 1 for details); (ii) cysteine which has a low mass and comprises sulfur; (iii) chloramphenicol which has a low mass and comprises chlorine; (iv) digoxigenin monodigitoxoside which has a medium mass and comprises only the elements CHNO; (v) 2-Chloro-$2^{\prime }$-deoxyadenosine-$5^{\prime }$-triphosphate which has a medium mass and comprises chlorine; and (vi) autoinducer-2 which has a low mass and contains boron. The isotopic fine structure of these substances is not detectable at this resolution and hence each simulated peak is a mixture of multiple peaks from the isotopic fine structure. We only include isotope peaks with an abundance of at least 0.01% of the abundance of the monoisotopic peak which results in isotope clusters of size 4, 5, 6, 6, 6, and 6 respectively.

For each isotope cluster, we calculate the minimal absolute mass error $\Delta m^{abs}$ in units of dalton and the minimal relative mass error $\Delta m^{ppm}$ in units of PPM which are required for a successful isotope cluster detection. The incorporation of a mass error is necessary because the mass differences between individual isotope peaks depend on the elemental composition and hence deviates from the default mass difference of ^13^C isotopes. It is possible to use only one of both parameters or a combination of both parameters to enable the detection of isotope clusters (see Equation (2) in Section 4.2).

We merge all six isotope clusters resulting in a single synthetic spectrum comprising 33 peaks. We apply the isotope detection routines ${\mathrm{IDR}}_{\mathrm{NewNoVal}}$ and ${\mathrm{IDR}}_{\mathrm{NewVal}}$ as described in Section 4.2 to the synthetic spectrum. We evaluate whether the isotope detection routines are able to assemble the original isotope clusters.

In Table 1 we show the results. We find that ${\mathrm{IDR}}_{\mathrm{NewNoVal}}$ is able to detect all six isotope clusters provided that a sufficiently large mass error is set (e.g., $\Delta m^{abs}=0.01$). In case of a smaller mass error (e.g., $\Delta m^{abs}=0.005$) we find that isotope clusters become split at isotope peaks which are dominated by the isotopes of sulfur, chlorine, or boron, i.e., the second isotope peak of substance (ii); the second and fourth isotope peak of substance (iii); the second isotope peak of substance (v); and the first isotope peak of substance (vi). We find that ${\mathrm{IDR}}_{\mathrm{NewVal}}$ is able to validate all but one isotope cluster. The first peak of the boron-containing substance (vi) is not included in the isotope cluster, because the abundance of this peak is too small relative to the space of biological substances of this mass. Hence, the excluded peak is assumed to be a potential hydrogen-loss. However, this isotope cluster can be correctly identified without validation or with specialized approaches [31].

## 3. Discussion

Aiming at the exhaustive detection and precise validation of isotope clusters we propose an additional targeted peak picking step with predicted isotope ROIs and the mass–specific validation of putative isotope clusters based on database statistics. Compromising between peak reliability and exhaustive detection we use a relaxed signal-to-noise of 6.25 threshold for predicted isotope ROIs and achieve an increase of +37.6% isotope peaks and +102.8% PPS. We use this relaxed signal-to-noise threshold by default in the freely available implementation of this algorithms in the R package xcms. The targeted peak picking with predicted isotope ROIs can easily be adapted in other tools such as *MZmine2* [38], *apLCMS* [39], and related approaches [40]. The validation of putative isotope clusters in combination with predicted isotope ROIs results in the highest number of correctly predicted molecular formulas and also the highest number of correct molecular formulas among the first three ranks. However, the ranks of correctly predicted molecular formulas were robust with respect to different approaches for peak picking and isotope cluster detection and it is challenging to improve upon. We exemplify the use of the proposed isotope detection routine with and without mass–specific isotope cluster validation and find that it is possible to detect substances with and without biologically unusual elements using an absolute mass error of 0.01 dalton. Consequently, we use this absolute mass error by default in the freely available implementation of these algorithms in the R package *CAMERA*.

The enhanced isotope cluster detection and validation presented in this work could improve the accuracy of substance quantification. All isotope peaks of one isotope cluster originate from the same substance and we point out that the consideration of a greater number of features from a certain substance—although small and noisy—reduces the technical variance in the data. In turn, this would enhance the precision and yield of comparative analyses, because a reduced data variance would not only improve calculated fold changes but would enable the statistically valid detection of smaller effect sizes. The slight improvement in molecular formula prediction could affect a considerable number of substances in case of metabolome-scale metabolite identification studies. Especially in untargeted metabolomics reliable hints for metabolite identification are urgently needed.

## 4. Materials and Methods

We present the methodology of the proposed approach and the used data for evaluation. Specifically, we describe (i) the targeted peak picking with predicted isotope ROIs; (ii) the detection and mass–specific validation of isotope clusters; (iii) the computation of isotope ratio quantiles; and (iv) two sets of mass spectrometry raw data.

### 4.1. Targeted Peak Picking with Predicted Isotope ROIs

A requirement for the prediction of isotope ROIs is a set of peaks that have been detected previously. This initial peak picking can be accomplished by one of the numerous peak picker which are available [1,18,38]. In untargeted approaches, these peak picker typically do not use any prior knowledge and we refer to this kind of peak picking as *traditional peak picking*. We propose the following approach for the targeted detection of isotope peaks. This approach is designed for liquid chromatography–high resolution mass spectrometry data and does not consider the isotopic fine structure available with ultrahigh resolution mass spectrometry.

**Figure 6:**
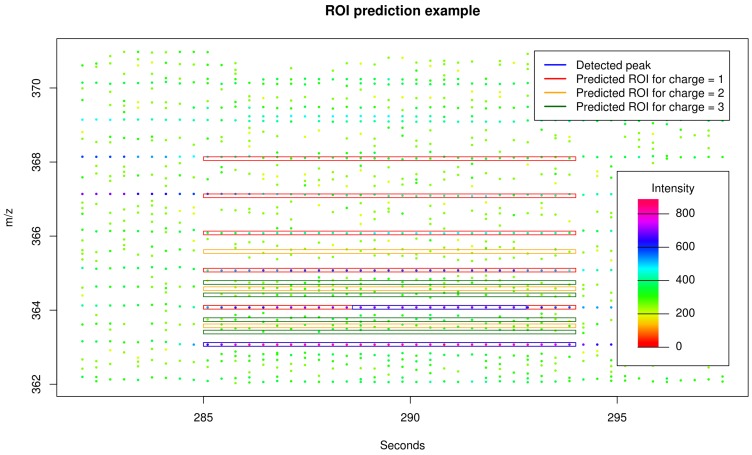
Exemplary section of LC-MS raw data. We mark two detected peaks from traditional peak picking in blue and 12 predicted isotope ROIs in red, orange, and green calculated on basis of the (monoisotopic) peak (apex *m*/*z* ≈363.075 dalton / retention time ≈291 seconds) given a maximum isotope number I=5 and a maximum charge state Z=3. Via prediction of isotope ROIs, we are able to expand the region of the already detected first isotope peak and to encompass the signals of the second, third, fourth, and fifth isotope peak. Here, the subsequent peak picking procedure will not find relevant signals for the predicted isotope ROIs corresponding to the charge states 2 (orange) and 3 (green) and will reject these accordingly.

Given a set of detected peaks from traditional peak picking, a maximum charge Z=3, and a maximum number of isotopes I=5 we predict putative isotope ROIs as follows. For each charge state z∈{1,...,Z} and for each isotope number i∈{1,...,I}, we compute the theoretical *m*/*z* distance to the monoisotopic peak

(1) $$d_{z,i}=\frac{i*\Delta m}{z},$$

where $\Delta m=\mathrm{mass}{(}^{13}\text{C})$ – $\mathrm{mass}{(}^{12}\text{C})$ ≈1.003355. We use Δm as an approximation for the mass difference between successive peaks in isotope clusters because the isotopic nuclide ^13^C has usually the largest impact on isotope clusters in biological samples. Other isotopic nuclides such as ^15^N, ^18^O, and ^34^S cause isotope peaks with mass differences which can only be discriminated from ^13^C-isotope peaks using mass spectrometers with resolution above 40,000 (in case of ions with an *m*/*z* of 500 dalton). For each peak detected by traditional peak picking we predict for each charge state *z* and for each isotope number *i* one putative isotope ROI. Each putative isotope ROI is composed of the retention time interval of the detected peak and the *m*/*z* interval of the detected peak shifted by $d_{z,i}$ as exemplified in Figure 6. An additional targeted peak picking is performed based on the set of predicted isotope ROIs using a relaxed signal-to-noise threshold ${\mathrm{snthr}}^{\prime }=\mathrm{snthr}*r/100$, where snthr is the signal-to-noise threshold for traditional peak picking and r∈{100,95,...,5}. Subsequently, the peak table from traditional peak picking and the peak table from the targeted peak picking on basis of putative isotope ROIs are merged and redundant peaks are removed.

For control purposes, we generate a set of noise ROIs given the set of predicted isotope ROIs as follows. To approximate the distribution of the predicted isotope ROIs in the *m*/*z* dimension and the retention time (RT) dimension, we calculate the minimum and maximum *m*/*z* and RT of the predicted isotope ROIs and use a uniform distribution in the calculated intervals of both dimensions. To approximate the distribution of peak widths in *m*/*z* and RT we calculate a histogram of peak widths in *m*/*z* relative to the peak *m*/*z* and a histogram of peak widths in RT. For each predicted isotope ROI we sample one new noise ROI which *m*/*z* and RT is uniformly drawn within the calculated ranges in *m*/*z* and RT and which peak width in *m*/*z* and RT is drawn from the calculated histograms. Subsequently, targeted peak picking is applied to the set of noise ROIs using a relaxed signal-to-noise threshold snthr’ analog to predicted isotope ROIs and the results from traditional peak picking and targeted peak picking on basis of noise ROIs are merged as before.

### 4.2. Detection and Mass–Specific Validation of Isotope Clusters

We propose an approach for the detection and validation of isotope clusters in liquid chromatography–high resolution mass spectrometry data which does not resolve the isotopic fine structure. In this approach we detect putative isotope clusters based on characteristic distances in the *m*/*z* dimension. We validate putative isotope clusters depending on the substance mass and we refer to this validation as *mass–specific validation*. We detect and validate isotope clusters given a set of coeluting features, a maximum charge Z=3, a relative *m*/*z* error in ppm $\Delta m^{ppm}$, and an absolute *m*/*z* error $\Delta m^{abs}$ as follows.

First, we detect putative isotope clusters. For each charge state z∈[1,Z], we mark all pairs of peaks $\left(p_{1},p_{2}\right)$ for which

(2) $$\delta_{z,p_{1},p_{2}}=||mass\left(p_{1}\right)-mass\left(p_{2}\right)\left|-\Delta m/z\right|\leq \max\left(\frac{mass\left(p_{1}\right)*\Delta m^{ppm}}{10^{6}},\Delta m^{abs}\right)$$

holds, where $\Delta m=\mathrm{mass}{(}^{13}\text{C})-\mathrm{mass}{(}^{12}\text{C})\approx 1.003355$ is the expected distance between two isotope peaks (cf. Section 4.1). For each charge state and for each peak *p*, we compute all putative isotope clusters $\left(p_{1},p_{2},...,p_{n}\right)$ for which $\delta_{c,p^{\prime },p^{\prime \prime }}$ holds for each successive pair of peaks $\left(p^{\prime },p^{\prime \prime }\right)$. We retain the putative isotope cluster with the maximum number of peaks and remove the peaks of this putative isotope cluster from the set of available peaks. We iteratively perform the last steps with the remaining peaks until there are no putative isotope clusters with at least two peaks left.

Second, we validate the set of putative isotope clusters which have been extracted previously depending on the monoisotopic mass. See Figure 3 for four cases which necessitate the following validation of putative isotope clusters. For each putative isotope cluster $\left(p_{1},p_{2},...,p_{n}\right)$ we examine the second to last peak $p^{\prime }\in \left(p_{2},...,p_{n}\right)$. For each peak $p^{\prime }$ we compute the ratio of the abundance of the monoisotopic peak $p_{1}$ and the abundance of peak $p^{\prime }$. Specifically, we compute the minimum and maximum ratio considering that the abundance estimates of both peaks are affected by the ubiquitous noise using an estimate of the signal-to-noise ratio of both peaks. If the computed interval of ratios does not overlap with the 99% confidence interval derived from the KEGG database for the current monoisotopic mass (mass window size 50) we split the putative isotope cluster. In this case we turn the peak $p^{\prime }$ into the new monoisotopic peak resulting in a new putative isotope cluster $\left(p^{\prime },...,p_{n}\right)$ which is validated as well. We retain all putative isotope clusters which comprise at least two peaks and consider these as validated isotope clusters.

### 4.3. Isotope Ratio Quantiles

We perform isotope statistics for each of the databases ChEBI, KEGG, KNApSAcK, LIPID MAPS, and PubChem as follows [32,33,34,35,36]. We iterate all compounds, compute the exact mass and the theoretical isotope cluster from the molecular formula, and record the ratio of the monoisotopic peak to the first to fifth isotope peak. We group all compounds by the exact mass in consecutive mass windows for each of the mass window sizes 10, 25, 50, 100, and 250 dalton to support different compromises between mass specificity and quantile robustness. For each mass window size, each mass window, and each isotope peak (1st–5th) we compute the isotope ratio for several *p*-quantiles, where $p\in \{5.0\times 10^{-6}$, 0.999995, $1.0\times 10^{-5}$, 0.99999, $5.0\times 10^{-5}$, 0.99995, $1.0\times 10^{-4}$, 0.9999, $5.0\times 10^{-4}$, 0.9995, 0.001, 0.999, 0.005, 0.995, 0.01, 0.99, 0.025, 0.975, 0.05, 0.95, 0.1, 0.9, 0.5}. For each mass window size and each isotope peak we record the isotope ratio in a matrix with one row for each *p*-quantile and one column for each mass window. We encapsulate the resulting data for each database, each mass window size, and each isotope peak in an R object of class *S4* named compoundQuantiles. This implementation supports a simple API for convenient retrieval of the data (see documentation of package *CAMERA* version 1.50.0 for details). Based on this implementation, it is also possible to compute isotope ratios amongst isotope peaks, e.g., the confidence interval of the isotope ratio between the third isotope peak and the fifth isotope peak for a given mass range.

### 4.4. Data Sets

#### 4.4.1. MM48

**Figure 7:**
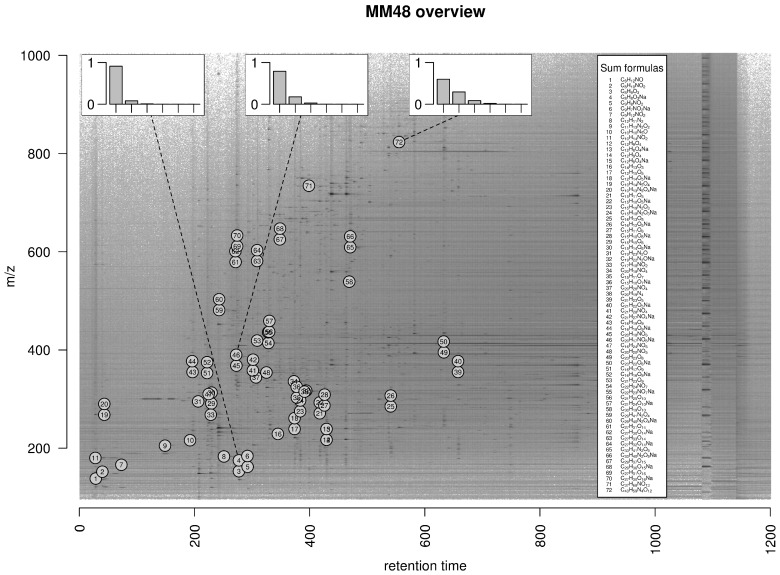
Overview of monoisotopic measurable ions in the MM48 data set. We plot the logarithmic raw data intensities in the dimensions mass-to-charge ratio (m/z) and retention time and mark the location of 72 monoisotopic ions which are measurable in at least six of eleven data sets. In case of three ions with exact mass 175.037, 390.095, and 823.413 dalton, we exemplarily plot the theoretical relative intensities of the monoisotopic peak and the first to fifth isotope peak in the insets at the top. The set of measurable ions spans a huge range in both dimensions with different isotope clusters constituting a diverse basis for validation purposes.

We perform a case study based on a gold standard data set comprising 11 LC-MS measurements (UPLC-ESI-QTOF-MS, positive mode) each of a solution of 48 known reference substances denoted as *MM48*. The raw data is available in MetaboLights [41] accession MTBLS381 in Supplementary Materials link. This set of compounds was also used in [24] and the measurements have been deposited in MetaboLights accession MTBLS188. We compile a ground truth of detectable ions as follows. First, we assume a set of three expected ions (${\left[M\right]}^{+}$, ${\left[M\;+\;H\right]}^{+}$, ${\left[M\;+\;\mathrm{Na}\right]}^{+}$) as well as isotope peaks up to the fifth isotope peak (i.e., ${\left[M\;+\;1\right]}^{+}$, ${\left[M\;+\;2\right]}^{+}$, ${\left[M\;+\;3\right]}^{+}$, ${\left[M\;+\;4\right]}^{+}$, and ${\left[M\;+\;5\right]}^{+}$ in case of the ${\left[M\right]}^{+}$ ion) for each compound and calculate the exact mass of these 18 molecular formulas (three ions each with an isotope cluster with six peaks); Second, we check the abundance of these ions in the 11 data sets and define all ions with a peak area of at least 1000 counts within a retention time interval of at most five seconds as measurable ions constituting the ground truth. Considering the set of ions which are measurable in at least six of 11 data sets, we detect 72 monoisotopic ions (see Figure 7), 63 isotope clusters with at least two ions, and 190 ions in total.

#### 4.4.2. Dilution Series

We perform a case study based on 40 LC-MS measurements (UPLC-ESI-QTOF-MS, positive mode), which is a subset of the data used in [24] and is available from the MetaboLights repository with accession MTBLS188. This set of measurements is composed of a dilution series varying the ratio of solution and leaf sample. Specifically, the ratio of solution and leaf sample is 0:100, 25:75, 50:50, and 75:25 in 10 data sets each. This experimental design implies a diverse range of cases in the data regarding the signal-to-noise ratio of peaks and constitutes the basis to test the detection of weak signals like isotope peaks.

## 5. Conclusions

We implemented the targeted peak picking with predicted isotope ROIs in combination with the *centWave* algorithm as part of the R package *xcms* in version 1.50.0 (functions findPeaks.centWaveWithPredictedIsotopeROIs and findPeaks.addPredictedIsotopeFeatures). We implemented the mass–specific validation of putative isotope clusters as part of the R package *CAMERA* in version 1.30.0 (function findIsotopesWithValidation).

## Figures and Tables

**Table 1:** Isotope cluster detection exemplified for six substances. We show the substance name, the sum formula, the mass of the monoisotopic peak and the first five isotope peaks (rounded to five digits), the mass difference to the monoisotopic peak (Δm, rounded to five digits), the relative peak intensity (Int., normalized to 100 and rounded to two digits), the absolute *m*/*z* error $\Delta m^{abs}$ and the relative *m*/*z* error in ppm $\Delta m^{ppm}$ for a successful isotope cluster detection ($\Delta m^{abs}$ is rounded to five digits and $\Delta m^{ppm}$ is rounded to one digit), whether the isotope cluster assignment using the isotope detection routine without isotope cluster validation ${\mathrm{IDR}}_{\mathrm{NewNoVal}}$ is successful or not (No val., “+”/“–”), and whether the isotope cluster assignment using the isotope detection routine with mass–specific isotope cluster validation ${\mathrm{IDR}}_{\mathrm{NewVal}}$ is successful or not (Val., “+”/“–”). ${\mathrm{IDR}}_{\mathrm{NewNoVal}}$ is able to detect the isotope clusters of all substances and ${\mathrm{IDR}}_{\mathrm{NewVal}}$ successfully validates the isotope clusters of all but one substance.

Substance Name	Sum Formula	Mass	Δm	Int.	$\Delta m^{\mathrm{abs}}$	$\Delta m^{\mathrm{ppm}}$	No Val.	Val.
Aspartic acid	C_4_H_7_NO_4_	133.037508		100.00	0.00191	14.3	+	+
		134.040468	1.00296	4.96			+	+
		135.041918	2.00441	0.93			+	+
		136.044728	3.00722	0.04			+	+
Cysteine	C_3_H_7_NO_2_S	121.019749		100.00	0.00895	73.9	+	+
		122.021976	1.00223	4.59			+	+
		123.016385	1.99664	5.05			+	+
		124.019165	2.99942	0.19			+	+
		125.018404	3.99866	0.03			+	+
Chloramphenicol	C_11_H_12_Cl_2_N_2_O_5_	322.012327		100.00	0.00913	28.4	+	+
		323.015369	1.00304	13.00			+	+
		324.009595	1.99727	66.20			+	+
		325.012562	3.00024	8.53			+	+
		326.007250	3.99492	11.54			+	+
		327.010016	4.99769	1.45			+	+
Digoxigenin monodigitoxoside	C_29_H_44_O_8_	520.303618		100.00	0.00078	1.5	+	+
		521.307027	1.00341	32.24			+	+
		522.309803	2.00619	6.70			+	+
		523.312531	3.00891	1.04			+	+
		524.315166	4.01155	0.13			+	+
		525.317742	5.01412	0.01			+	+
2-Chloro-2’-deoxyadenosine-5’-triphosphate	C_10_H_15_ClN_5_O_12_P_3_	524.961858		100.00	0.00817	15.6	+	+
		525.964411	1.00255	13.30			+	+
		526.959596	1.99774	35.41			+	+
		527.962023	3.00017	4.63			+	+
		528.963673	4.00182	1.11			+	+
		529.966017	5.00416	0.12			+	+
Autoinducer-2	C_5_H_10_BO_7_	192.055590		24.37	0.00689	35.9	+	–
		193.052059	0.99647	100.00			+	+
		194.055706	2.00012	6.13			+	+
		195.056530	3.00094	1.59			+	+
		196.059851	4.00426	0.09			+	+
		197.060963	5.00537	0.01			+	+

## Supplementary Materials

The following are available online at www.ebi.ac.uk/metabolights/MTBLS381, 11 MM48 raw data files used for performance evaluation in the manuscript.

## Appendix A. Isotope Cluster Detection and Validation: Extended Results

We compare the proposed isotope detection routine with mass–specific isotope cluster validation (${\mathrm{IDR}}_{\mathrm{NewVal}}$) against the isotope detection routine without isotope cluster validation (${\mathrm{IDR}}_{\mathrm{NewNoVal}}$), the isotope detection routine implemented in the *AStream* package (${\mathrm{IDR}}_{\mathrm{AStream}}$) [29], the isotope detection routine implemented in the *CAMERA* package (${\mathrm{IDR}}_{\mathrm{CAMERA}}$) [24], and the isotope detection routine implemented in the *mzMatch* package (${\mathrm{IDR}}_{\mathrm{mzMatch}}$) [30].

We evaluate the performance of the isotope cluster detection and validation described in Section 4.2 on a dilution series experiment with 40 LC-MS measurements described in Section 4.4. We quantify the performance using the performance measures (i) number of detected peaks; (ii) number of detected isotope peaks; (iii) number of detected isotope clusters; and (iv) isotope coverage, i.e., the ratio of the number of detected isotope peaks and the number of all detected peaks. We compute each performance measure as a function of the relaxed signal-to-noise threshold ${\mathrm{snthr}}^{\prime }\;\in \;\left\{100,95,...,5\right\}\;\%\;*\;\mathrm{snthr}$, where snthr=25 is the signal-to-noise threshold of the traditional peak picking step. In the Section 2.3 we show an excerpt of these results, i.e., we present the results for each isotope detection routine with predicted isotope ROIs relative to the results of ${\mathrm{IDR}}_{\mathrm{CAMERA}}$ without predicted isotope ROIs in Figure 4.

In Figure A1 we show the performance measures for ${\mathrm{IDR}}_{\mathrm{NewVal}}$, ${\mathrm{IDR}}_{\mathrm{NewNoVal}}$, ${\mathrm{IDR}}_{\mathrm{AStream}}$, ${\mathrm{IDR}}_{\mathrm{CAMERA}}$, and ${\mathrm{IDR}}_{\mathrm{mzMatch}}$.

## Appendix B. Prediction of Molecular Formulas From Isotope Clusters

In order to study to which degree the proposed approach is capable of improving the detection and validation of isotope clusters, we test the quality of predicted molecular formulas. The prediction of molecular formulas is an important step towards the identification of substances and can be done automatically on the basis of isotope clusters. We use 11 LC-MS measurements with 48 known compounds and select a set of 72 ions. We predict for each ion a list of ranked molecular formula candidates using SIRIUS and evaluate the rank of the correct molecular formula [3].

We evaluate the performance of predicted isotope ROIs described in Section 4.1 and the isotope detection routine with mass–specific isotope cluster validation described in Section 4.2 on 11 LC-MS measurements of known compounds described in Section 4.4 using predicted molecular formulas from SIRIUS as described in the Appendix D.4. We quantify the performance using the number of compounds with a certain rank averaged over all measurements. If the proposed approaches increase the quality of detected isotope clusters, then the rank of the predicted molecular formulas should decrease and be ranked first in the ideal case. We compare different combinations of two peak picking approaches and five isotope detection routines, namely (iA) the traditional peak picking and (iB) the traditional peak picking in combination with targeted peak picking with predicted isotope ROIs (see Section 4.1) and (iiA) the isotope detection algorithm from *AStream*; (iiB) the isotope detection algorithm from *mzMatch*; (iiC) the isotope detection algorithm from *CAMERA*; (iiD) the proposed isotope detection algorithm without isotope cluster validation; and (iiE) the proposed isotope detection algorithm with mass–specific isotope cluster validation resulting in ten combinations of algorithms (see Section 4.2 and the Appendix D). In Table B1 we show the ranks of the predicted molecular formulas for ten algorithms averaged over 11 data sets.

**Table B1:** Molecular formula prediction from isotope clusters. Using SIRIUS we predict molecular formulas from isotope clusters which have been detected using different algorithms. In the first column we indicate whether we use targeted peak picking with predicted isotope ROIs (’+’) or not (’−’) and in the second column we indicate the isotope detection algorithm (${\mathrm{IDR}}_{\mathrm{AStream}}$ for the algorithm implemented in R package *AStream*, ${\mathrm{IDR}}_{\mathrm{CAMERA}}$ for the algorithm implemented in R package *CAMERA*, ${\mathrm{IDR}}_{\mathrm{mzMatch}}$ for the algorithm implemented in R package *mzMatch*, ${\mathrm{IDR}}_{\mathrm{NewNoVal}}$ for the proposed isotope detection algorithm without isotope cluster validation, and ${\mathrm{IDR}}_{\mathrm{NewVal}}$ for the proposed isotope detection algorithm with mass–specific isotope cluster validation). We specify the number of ions with a molecular formula on rank 1, on rank 2, on rank 3, between rank 4 and rank 10, on a rank above 10, the number of ions which molecular formula is not among the top 1000 candidates (’No rank’), and the number of ions which have not been detected during peak picking (’No peak’). We arranged the isotope detection algorithms by the number of ions with molecular formula on rank 1.

Predicted Isotope ROIs	Isotope Detection Algorithm	Rank 1	Rank 2	Rank 3	3 < Rank ≤ 10	Rank > 10	No Rank	No Peak
−	${\mathrm{IDR}}_{\mathrm{mzMatch}}$	48.82	11.55	1.18	3.36	0	4.64	2.45
+	${\mathrm{IDR}}_{\mathrm{mzMatch}}$	48.18	12	1.18	3.36	0	4.82	2.45
−	${\mathrm{IDR}}_{\mathrm{NewNoVal}}$	49.09	10.91	0.91	1.55	0	7.09	2.45
+	${\mathrm{IDR}}_{\mathrm{NewNoVal}}$	49.36	11.18	0.73	1.64	0	6.73	2.36
−	${\mathrm{IDR}}_{\mathrm{AStream}}$	52.82	11.27	1.09	1.82	0	2.55	2.45
+	${\mathrm{IDR}}_{\mathrm{AStream}}$	53.27	11.55	0.55	1.91	0	2.36	2.36
−	${\mathrm{IDR}}_{\mathrm{CAMERA}}$	53.73	10.27	0.82	1.55	0	3.18	2.45
+	${\mathrm{IDR}}_{\mathrm{CAMERA}}$	52.82	11	0.64	1.64	0	3.55	2.36
−	${\mathrm{IDR}}_{\mathrm{NewVal}}$	53.82	11.09	1	1.55	0	2.09	2.45
+	${\mathrm{IDR}}_{\mathrm{NewVal}}$	54.09	11.36	0.73	1.64	0	1.82	2.36

## Appendix C. Isotope Cluster Statistics: Full Quantile Set for PubChem

In Figure C1 we depict all computed quantiles and the resulting symmetric confidence intervals of the isotope ratio of the monoisotopic peak to the first isotope peak for the database PubChem with a mass window size equal to 50 dalton. See Section 4.3 for a detailed description of the database statistics.

## Appendix D. Software Versions and Processing Parameters

Tools versions, used functions, and parameters of *xcms/CAMERA*, *AStream*, *mzMatch*, and *SIRIUS* are given subsequently.

### Appendix D.1. xcms/CAMERA

We use the R package *xcms* version 1.44.0 [23] and the R package *CAMERA* version 1.27.0 [24] for peak picking using *centWave* [1], the grouping of features into pseudospectra, and the detection of isotope clusters. We processed the raw data of each LC-MS measurement individually as follows. We performed peak picking with the *centWave* algorithm with parameters peakwidth=(5,12), prefilter=(2,200), ppm=10, and snthr=25. We use a signal-to-noise ratio of 25, because it has been shown that this ratio yields reliable molecular formula predictions from mass spectrometry data [42]. Subsequently, we group detected peaks by retention time into pseudospectra-groups using function *groupFWHM* with perfwhm=1 and standard parameters and detect isotope clusters using function *findIsotopes* with intensityValue=’intb’ and standard parameters.

### Appendix D.2. AStream

We use the R package *AStream* version 2.0 [29] for the detection of isotope clusters. We import the peaks which have been detected using *xcms* into the *AStream* datalist structure. We apply the function data.norm with the parameters mz.tol=0.005 (the mean *m*/*z* error for ppm=10 as used in *xcms* and *mzMatch*) and we detect isotope clusters using function isotope.search with the parameter mz.tol=0.005. In a postprocessing step we remove contradictory isotope annotations, i.e., if (i) peak B is annotated as [M + 1] isotope peak of peak A and (ii) peak C is annotated as [M + 2] isotope peak of peak A and (iii) peak C is annotated as [M + 1] isotope peak of peak B; then we remove annotation (iii).

### Appendix D.3. mzMatch

We use the R package *mzmatch.R* version 2.0-13 [30] for the detection of isotope clusters. We import the peaks which have been detected using *xcms* via the peakML file format used by *mzMatch* using the function PeakML.xcms.write.SingleMeasurement with the parameters writeRejected=TRUE, ppm=10, addscans=0, and ApodisationFilter=FALSE. We convert this data using function mzmatch.ipeak.Combine and we detect isotope clusters using function mzmatch.ipeak.sort.RelatedPeaks with the parameters ppm=10 and rtwindow=50. In a postprocessing step we remove all isotope clusters with gaps, i.e., the isotope cluster with monoisotopic peak [M] and isotope peak [M + 2] without the [M + 1] isotope peak is considered non-evaluable and removed from the output. Approximately 10% of the isotope annotations are removed in this way.

### Appendix D.4. Prediction of Molecular Formulas Using SIRIUS

We predict ranked candidate lists from isotope clusters using command–line SIRIUS [3] version 3.1.3. We use the parameters –elements=CHNOPS, –isotope=score, –candidates=1000, –ppm−max=10, and –profile=qtof and give the ion species (–ion), the monoisotopic *m*/*z* (–mz), the (*m*/*z*, intensity) pairs (into intensity from *xcms*; –ms1), and an empty MS/MS spectrum (–ms2) as input. We rank the resulting candidate lists according to the tree score and select the rank of the correct molecular formula.
